# Quantifying the Power and Precision of QTL Analysis in Autopolyploids Under Bivalent and Multivalent Genetic Models

**DOI:** 10.1534/g3.119.400269

**Published:** 2019-04-29

**Authors:** Peter M. Bourke, Christine A. Hackett, Roeland E. Voorrips, Richard G. F. Visser, Chris Maliepaard

**Affiliations:** * Plant Breeding, Wageningen University & Research, Droevendaalsesteeg 1, P.O. Box 386, 6700 AJ Wageningen, The Netherlands; † Biomathematics and Statistics Scotland, Invergowrie, Dundee DD2 5DA, UK

**Keywords:** Quantitative Trait Locus (QTL) analysis, autopolyploid, double reduction, Bayesian Information Criterion (BIC), genotypic information coefficient (GIC)

## Abstract

New genotyping technologies, offering the possibility of high genetic resolution at low cost, have helped fuel a surge in interest in the genetic analysis of polyploid species. Nevertheless, autopolyploid species present extra challenges not encountered in diploids and allopolyploids, such as polysomic inheritance or double reduction. Here we investigate the power and precision of quantitative trait locus (QTL) analysis in outcrossing autopolyploids, comparing the results of a model that assumes random bivalent chromosomal pairing during meiosis to one that also allows for multivalents and double reduction. Through a series of simulation studies we found that marginal gains in QTL detection power are achieved using the double reduction model when multivalent pairing occurs. However, when exploring the effect of variable genotypic information across parental homologs, we found that both QTL detection power and precision require high and uniform genotypic information contents. This effect far outweighed considerations regarding bivalent or multivalent pairing (and double reduction) during meiosis. We propose that autopolyploid QTL studies be accompanied by both marker coverage information and per-homolog genotypic information coefficients (GIC). Application of these methods to an autotetraploid potato (*Solanum tuberosum* L.) mapping population confirmed our ability to locate and dissect QTL in highly heterozygous outcrossing autotetraploid populations.

Autopolyploid species, characterized by having more than two homologous copies of each chromosome, present a number of challenges to genetic research not present in diploids or allopolyploids (which are essentially already diploidized, genetically-speaking). They have therefore been somewhat left behind when it comes to tools and methods for their genetic analysis. Among these challenges are the complexity of modeling polysomic inheritance and the occurrence of double reduction (the phenomenon whereby a particular segment of a parental chromatid and its recombinant “sister” copy migrate to the same gamete, which can only occur if multivalent pairing structures are formed and maintained through meiosis 1 (Haldane 1930; Mather 1935)). However, we are now at a stage where the availability and low cost of genotyping tools (based on single nucleotide polymorphisms, or SNP markers) are making the analysis of autopolyploids not just feasible, but of practical importance to crop breeders, increasing the need for both the methods and the tools to conduct these analyses, as well as knowledge on how best to apply these methods.

Breeders and researchers are often interested in knowing the genetic architecture of important traits, for example: 1. whether they are mono-, oligo- or polygenic; 2. where the quantitative trait loci (QTL) influencing the trait lie on the genome; 3. from which specific parental homologous chromosome (which we term “homolog”) the favorable alleles originate; 4. whether these alleles exhibit additive or dominant gene action; 5. whether they interact with alleles at other loci, or with the environment. Up to now, approaches such as QTL mapping in bi-parental populations or genome-wide association studies have been proposed, although not always addressing all of the above points. More recently, genomic selection has been advocated as a powerful method to increase genetic gain, without necessarily needing an understanding of the genetic architecture (Meuwissen *et al.* 2001; Slater *et al.* 2016). For quantitative traits with hundreds or thousands of causative genes, this is likely to be more appropriate in breeding programs. However, for traits with a few major causative loci, QTL mapping remains a viable option that additionally offers the promise of both understanding the genetics underlying the trait (including the possibility of finding the underlying genes) while also facilitating selection for it through marker-assisted selection.

QTL mapping in autopolyploids (particularly autotetraploids) has evolved in the last 20 years to keep pace with changes in genotyping technologies. Approaches have been developed for both co-dominant and dominant marker systems, and range from simplified models that only consider bivalent pairing to more complex models that also include double reduction. However, there has been almost no investigation into the applicability or advantages of different models. Despite this, it is often asserted that models that ignore double reduction are *a priori* inferior to those that include it (Luo *et al.* 2004; Luo *et al.* 2006). More complete models of polysomic inheritance that include double reduction are often developed under the assumption of completely-informative marker systems (*e.g.*, (Li *et al.* 2010; Xie and Xu 2000; Xu *et al.* 2013)), thereby avoiding the statistical complexities imposed by partially-informative markers (such as dosage-scored SNP markers). In fact, it is quite telling that the only publically-available tools for QTL analysis in autopolyploids have adopted the simplifying assumption of random bivalent pairing (*e.g.*, TetraploidMap (Hackett and Luo 2003; Hackett *et al.* 2007) and TetraploidSNPMap (Hackett *et al.* 2017)), whereas more complete QTL model descriptions remain unimplemented or unavailable.

Recently, a method to reconstruct inheritance probabilities (or identity-by-descent (IBD) probabilities) under both bivalent and multivalent pairing models in autotetraploid F_1_ populations was implemented in the software package TetraOrigin (Zheng *et al.* 2016). IBD probabilities describe the relative likelihood of inheritance of all possible combinations of parental alleles at a locus, for all offspring in a mapping population (Figure 1). They replace marker genotypes by pooling information from neighboring markers together (following a Markov chain procedure), essentially transforming bi-allelic markers into probabilistic multi-allelic markers. They are therefore very powerful as a replacement for single marker genotypes in QTL analyses. Using polyploid mapping populations simulated by PedigreeSim (Voorrips and Maliepaard 2012) and the IBD probabilities generated using the TetraOrigin algorithm, we investigated QTL mapping in autotetraploids and autohexaploids, estimating QTL detection power and precision, the effect of double reduction and multivalent pairing (while comparing models that both ignore and include double reduction), the impact of population size, trait heritability and marker distribution, and the differences in QTL detection and diagnostic power (*i.e.*, correctly predicting the QTL position as well as the composition and phase of the QTL alleles) between simple or more complex QTL segregation types and different modes of action (additive or dominant). We also examined the well-studied traits of plant maturity (earliness) and flesh color in a bi-parental tetraploid potato population to further illustrate our findings, comparing the QTL locations to the physical positions of candidate genes underlying these loci. Although we restricted our simulations to autotetraploids and autohexaploids (the most commonly-encountered autopolyploid levels), double reduction is a phenomenon that can occur at all higher ploidies, and therefore we expect our results to be broadly applicable.

**Figure 1:**
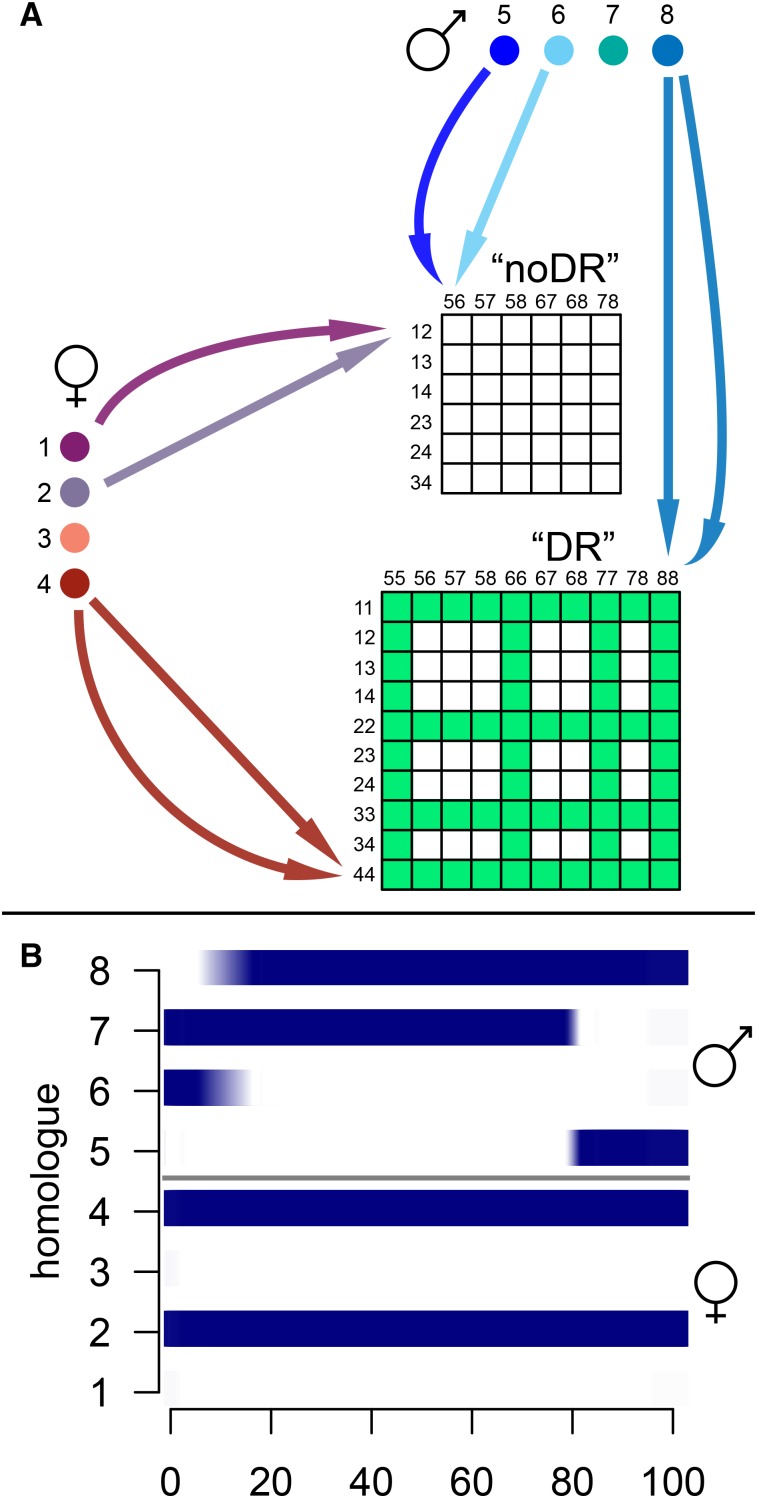
Tracking the inheritance of parental alleles in an autotetraploid. **A.** There are 36 possible combinations of parental alleles at a locus in each tetraploid offspring from a bivalent-only model (“noDR”). When double reduction (“DR”) is also considered, 64 extra genotype classes arise (shown in green), leading to a total of 100 possible allelic configurations at each locus. **B.** Visualization of “haplotypic” IBD probabilities in an individual offspring for one chromosome, showing the inheritance of homologs 2 and 4 (maternal), and segments of homologs 5 – 8 (paternal), with recombination break-points identified. Darker blue colors indicate a higher probability of inheritance. X-axis scale is in centiMorgans.

One important aspect of QTL mapping that remains conspicuously absent from most published QTL studies in both diploid and polyploid species is the topic of information content. Originally it was noted that “marker information content” could adversely affect the estimated position of a QTL if markers of variable informativeness were located near a QTL (Knott and Haley 1992). Increasing information content was found to lead to an increased test statistic, which could bias the location of a QTL peak (Knott *et al.* 1997; Knott and Haley 1992). Other authors have proposed alternative measures of information content than that of Knott and Haley, such as one based on Shannon’s information content (Reyes-Valdés and Williams 2005). In autopolyploid species the issue of information content is arguably even more important than in diploids, as information content generally varies between homologs. We prefer to use the term “genotypic information coefficient” (Van Ooijen 2009; van Ooijen 1992) as it avoids confusion when dealing with IBD probabilities, which are multi-point estimates of homolog transmission probabilities and not the marker genotypes themselves. In this study we also extend the definition of the genotypic information coefficient (GIC) to autopolyploids and explore its usefulness in predicting QTL detection power and precision.

## Materials and Methods

**Figure 2:**
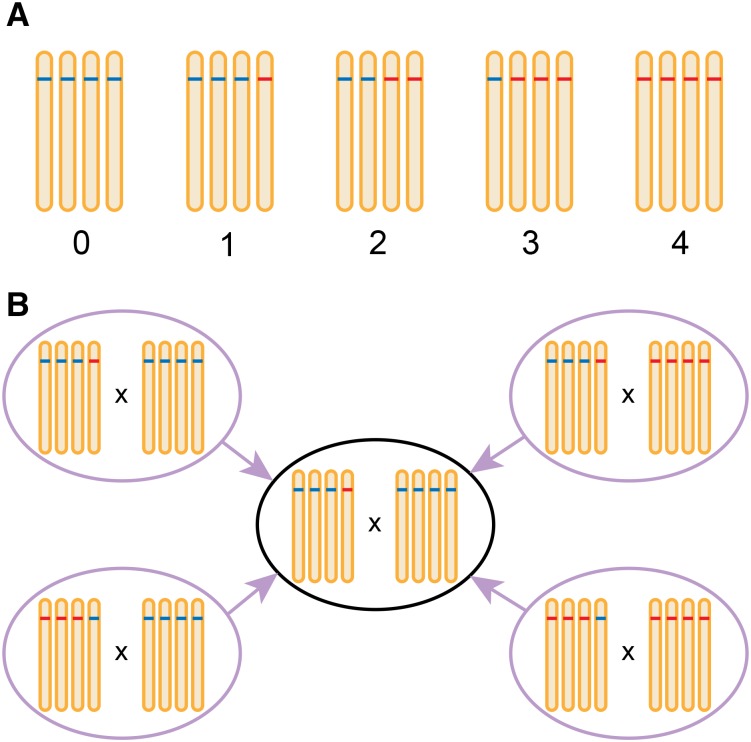
Marker dosage considerations in an autotetraploid. **A.** In an autopolyploid there are *ploidy* + 1 distinct marker dosage classes at a bi-allelic marker, ranging from 0 copies to *ploidy* copies of the alternative allele. Here, dosage is assigned based on counts of the alternative (red) allele. **B.** Re-coding (“converting”) markers reduces the number of marker types to consider as well as helping visualizations of marker alleles by only showing segregating alleles. Here, four different possible simplex x nulliplex markers (1x0, 1x4, 3x0, 3x4) are converted to their most fundamental form (1x0).

Bi-parental tetraploid F_1_ mapping populations were simulated based on previously-published genetic maps of tetraploid potato developed from dosage-scored SNP markers (Hackett *et al.* 2013), while the hexaploid F_1_ populations were based on a randomly-generated set of marker positions. In polyploids, marker dosages correspond to the allele counts of the “alternative” allele (as opposed to the “reference” allele) of a bi-allelic SNP marker. In heterozygous autotetraploids the possible dosages are 0, 1, 2, 3 and 4 (Figure 2.a), with a marker being defined by its maternal and paternal dosages (*e.g.*, 1x0 means a dosage of 1 in parent 1 (the mother) and a dosage of 0 in parent 2 (the father)). There are nine “fundamental” marker segregation types to consider in an autotetraploid cross, namely 1x0, 0x1, 2x0, 0x2, 1x1, 1x3, 1x2, 2x1 and 2x2, rising to 19 in an autohexaploid, to which all other marker types can be converted without loss or distortion of linkage information (Figure 2.b) (Bourke *et al.* 2016). For convenience, we often refer to simplex x nulliplex or “SxN” markers to indicate both 1x0 or 0x1 markers (or indeed QTL). Similarly, duplex x nulliplex (DxN) implies either 2x0 or 0x2 (and SxS = 1x1).

Potato chromosomes 1 and 12 of Hackett *et al.* (2013) displayed contrasting levels of marker coverage (Supplementary Figure S1) and were therefore used to study different aspects. We used potato chromosome 1, with its uneven marker distribution, for the investigations into the genotypic information coefficient (GIC), while chromosome 12 was used for the power analysis (Table 1). Unless otherwise stated, all statistical analyses and visualizations were performed using the R statistical computing environment version 3.3.2 (R Core Team 2016).

**Table 1:** Details of parameter levels used in the simulation study

Simulation	q	Pop.	h^2^	QTL seg.	QTL action	QTL pos.	QTL model
**Tetraploid Potato chm. 1**	0	200	0.1	SxN	additive	random	noDR
**(GIC study)**	0.2	400	0.2	DxN	dominant		DR
	0.5			SxS			
**Tetraploid**	0	200	0.1	SxN	additive	14 cM	noDR
**Potato chm. 12**	0.1	400	0.2	DxN	dominant	49 cM	DR
	0.2			SxS			
	0.3						
	0.4						
	0.5						
**Hexaploid**	0	200	0.1	random	additive	random	noDR
	0.5		0.7		dominant		DR
	1.0						

The phased genetic linkage maps of potato chromosomes 1 and 12 from Hackett *et al.* (2013) were used in the simulation of genotyped tetraploid populations with PedigreeSim, while the hexaploid map was randomly generated. For each scenario, all possible parameter combinations mentioned here were used, resulting in 144 separate parameter combinations for potato chm. 1, 576 for potato chm. 12 and 24 for the hexaploid populations.

Chm. = chromosome; q = rate of quadrivalent formation (specified in PedigreeSim .chrom file), where 0 = no quadrivalents and 1 = only quadrivalents; Pop. = population size of F_1_ population; h^2^ = (broad-sense) trait heritability; QTL seg. = QTL segregation type, given as the maternal x paternal dosage counts of the favorable QTL allele. The codes SxN refers to 1x0 and 0x1 QTL (single copy of the favorable allele in one of the parents), DxN refers to 2x0 and 0x2 QTL and SxS refers to 1x1 QTL; QTL pos. = genetic position of the QTL. This was either random or confined to a telomeric (14 cM) *vs.* centromeric (49 cM) position on potato chm. 12; QTL model = model used in QTL analysis, either with double reduction (DR) or not (noDR).

### QTL detection model

QTL analysis was performed using a weighted regression of the homolog main effects, weighted by the IBD genotype probabilities (Hackett *et al.* 2014). The QTL model for autotetraploids described by Hackett *et al.* (2013, 2014), derived from the earlier work of Kempthorne (Kempthorne 1957), can be written as:

$$Y=\mu^{’}+\alpha_{2}X_{2}+\alpha_{3}X_{3}+\alpha_{4}X_{4}+\alpha_{6}X_{6}+\alpha_{7}X_{7}+\alpha_{8}X_{8}+\varepsilon \;$$

having taken the constraints $X_{1}+X_{2}+X_{3}+X_{4}=2$ and $X_{5}+X_{6}+X_{7}+X_{8}=2\;$into account (*i.e.*, we drop the terms *X_1_* and *X_5_* to remove collinearity between model terms). Here, *Y* corresponds to the trait values, *X_i_* the indicator variables for the presence / absence of a particular parental homolog (1-4 for parent 1, 5-8 for parent 2), $\mu^{’}$ the intercept and ε the residual term. Hackett *et al.* (2014) describe this as the “additive” model, and used the probabilities of the possible genotypes as weights in a regression using the above model form (Hackett *et al.* 2014; Kempthorne 1957). The corresponding model for an autohexaploid is:

$$Y=\mu^{’}+\alpha_{2}X_{2}+\alpha_{3}X_{3}+\alpha_{4}X_{4}+\alpha_{5}X_{5}+\alpha_{6}X_{6}+\alpha_{8}X_{8}+\alpha_{9}X_{9}+\alpha_{10}X_{10}+\alpha_{11}X_{11}+\alpha_{12}X_{12}+\varepsilon \;$$

again, having taken the constraints $X_{1}+X_{2}+X_{3}+X_{4}+X_{5}+X_{6}=3$ and $X_{7}+X_{8}+X_{9}+X_{10}+X_{11}+X_{12}=3$ into account.

The software package TetraOrigin (Zheng *et al.* 2016) can determine IBD probabilities in autotetraploid populations under both bivalent and multivalent pairing models. We applied TetraOrigin (run on Mathematica version 10 (Wolfram Research Inc. 2014)) with input files derived from the integrated linkage maps and dosage output of PedigreeSim. Both bivalent_decoding options (False / True) were run to generate IBD probabilities under both a model that allowed for double reduction (DR) and one that did not (noDR), visualized in Figure 1.a. The other parameter settings used were parental dosage error probability (epsF) = 0, offspring dosage error probability (eps) = 0.001, and parental bivalentPhasing = True (*i.e.*, assuming purely bivalent pairing predominates to determine parental marker phase, for computational efficiency). The TetraOrigin algorithm is generalisable to all even ploidy levels (Zheng *et al.* 2016), although for simplicity we only ran the latter step of offspring IBD estimation for hexaploids using the parental marker phasing from the simulated linkage maps as input. The IBD probabilities at the marker positions were used to fit splines (using the smooth.spline function in R (R Core Team 2016)) from which re-normalized probabilities were interpolated at a 1 cM grid of positions (using the predict function in R) for subsequent QTL analysis.

We first applied this approach using the noDR IBD probabilities as weights (which we term the “no double reduction” (noDR) model, where all $X_{i}$ = 0 or 1). This is identical to the approach used by Hackett *et al.* (2014) in their work on QTL analysis in tetraploid potato.

However, the IBD probabilities generated by TetraOrigin *without* the constraint of bivalent pairing can also be used as weights in a similar fashion, although the indicator variable matrix *X* must be modified accordingly (Supplementary File S1). We termed this the “DR” model, which allows for the possibility of genotypes resulting from double reduction, *i.e.*, $X_{i}$ are no longer constrained to equal 0 and 1, but rather $X_{i}$ = 0, 1 or 2.

The “logarithm of odds ratio” (LOD) score for the regression was calculated using the formula

$$LOD=\frac{N}{2}\;\log_{10}\left(\frac{RSS_{0}}{RSS_{1}}\right)$$

where *N* is the population size, *RSS_0_* is the residual sum of squares under the null hypothesis of no QTL ($RSS_{0}=\sum_{i}{\left(y_{i}-\bar{y}\right)}^{2}$ for trait values $y_{i}$ and overall trait mean $\bar{y}$), and *RSS_1_* is the residual sum of squares from the regression model (Broman *et al.* 2003). A chromosome-wide QTL scan was performed at 1 cM intervals and the LOD score recorded at each position.

Significance thresholds were determined through permutation tests (Churchill and Doerge 1994), with each of the 1000 simulated phenotype sets per parameter set (10 populations × 100 phenotypes) permuted once before recording the maximum LOD score from the chromosome-wide scan (*i.e.*, recording 1000 maxima). This generated approximate experiment-wise LOD thresholds by taking the 0.95 quantile of the sorted LOD values. A QTL was declared detected if the significance at the QTL position exceeded the significance threshold. Because the true positions of most QTL were not positioned exactly at the grid of 1 cM positions tested, approximate LOD scores were interpolated at the QTL positions (and used to derive QTL detection rates).

### GIC study – potato chromosome 1

For each set of population parameters (all possible combinations of population size (Pop.) and rate of quadrivalent formation (*q*)) we simulated 10 separate populations using PedigreeSim and the phased linkage map of chromosome 1 from Hackett *et al.* (2013) for the phased parental marker positions and dosages (visualized in Supplementary Figure S1, left-hand side). Each simulated individual carried a single chromosome. For each population, we generated 100 phenotype sets for all possible combinations of the factors heritability (*h^2^*), QTL segregation type (QTL seg.) and QTL action (Table 1). The phenotype of the *i*^th^ individual ($P_{i}$) with QTL dosage $d_{i}$ was randomly sampled from a Normal distribution according to:

$$P_{i}∼N(\mu +Q*d_{i},\sigma_{e}^{2})$$

where μ = 10, Q = 1. The environmental variance $\sigma_{e}^{2}=\left(\frac{1-h^{2}}{h^{2}}\right)\sigma_{g}^{2}$ was determined by first calculating the genotypic variance $\sigma_{g}^{2}$ across the whole population given the individual QTL dosages (in the case of a dominant QTL these were taken as a dosage of 0 and 1 only). Offspring QTL genotypes were derived from the .hsa and .hsb output files of PedigreeSim (which provide the exact location and origin of recombination points along offspring homologs). Both the position (to 0.01 cM accuracy) and the configuration of the QTL (*i.e.*, from which parental homologs the various QTL alleles originate) were randomized for each phenotype set.

The GIC values for homolog *j* at a particular locus were determined as follows:

$$GIC_{j}=1-\frac{4}{N}\sum_{n=1}^{N}\pi_{n}(1-\pi_{n})\;$$

using the noDR IBD probabilities, where $\pi_{n}$ is the probability of inheriting homolog *j* in individual *n* at this locus (derived in Appendix 1). GIC values were calculated at all 1 cM splined positions used in the QTL scan. We considered the extension of the GIC to include the case of double reduction, but found a homolog-specific GIC was no longer easily defined when an offspring can inherit more than one copy of part of a particular homolog. Note that this definition of GIC is independent of ploidy level.

To better understand the relative importance of GIC on the power of QTL detection, we applied a generalized linear model (GLM) (using a Binomial model with logit link) using the following model:

$$logit(p)=q+Pop+h^{2}+QTLseg+QTLact+GIC$$

where *p* is the probability of detecting the QTL, and the explanatory variables are *q* (rate of multivalent formation), *Pop* (Population size), *h^2^* (heritability), *QTLseg* (QTL segregation type), *QTLact* (mode of QTL action, either additive or dominant) and *GIC* (the product of per-homolog GIC values underlying the QTL alleles). We confirmed these results by also using the LOD score at the QTL position as the response variable (corrected for variable significance thresholds by subtracting the thresholds from the LOD scores first).

To understand the influence of GIC on the detection of more complex QTL segregation types, we categorized the per-homolog GIC as either high (H) or low (L) using a threshold of both 0.9 and 0.95 for high GIC (so for example in the former, Low GIC < 0.9 and High GIC ≥ 0.9). These cut-offs were empirically-chosen to divide the data in a relatively balanced manner between high and low GIC. A DxN or SxS QTL could then be categorized as either LL, LH, HL or HH, depending on the underlying GIC at each of the alleles with positive effect. For each parameter set we compared the power of detection of LL, LH / HL (since both have one low and one high-GIC allele they were grouped together) and HH QTL.

Finally, we performed a small investigation of the effect of founder haplotype on the GIC measure using populations simulated from a founder haplotype pool of 5, 10 or 20 haplotypes, described in Supplementary File S2.

### QTL power analysis – potato chromosome 12

We were primarily interested in understanding the factors that influence the power and precision of a QTL analysis in autopolyploid populations (with the influence of double reduction of particular interest). Here we define detection power as the rate at which a simulated QTL position has an associated LOD score that exceeds the LOD significance threshold. This is a more conservative definition of power than the alternative definition (which considers the rate of significant peaks detected). We also looked at whether simulated QTL fell within the LOD-1 and LOD-2 support intervals (the full range of positions with a LOD score whose difference to the maximum LOD is less than one, or two, respectively), which is yet another indication of QTL detection power. The precision of a QTL analysis was gauged in a number of ways – as the distance (in centiMorgans) between the QTL position and the peak LOD score, but also in how precisely the QTL configuration and mode of action was predicted.

The simulations using chromosome 12 were similar to those of chromosome 1 with some differences (Table 1). Six different rates of quadrivalent formation were tested (*q* = 0, 0.1, ..., 0.5) and for each set of population-wise parameters, 50 separate populations were simulated. For each simulated population, 50 sets of phenotypes were generated as described above, except that the position of the QTL was confined to two positions, namely 14 cM (telomeric) and 49 cM (centromeric). The choice of these positions was not arbitrary: they were chosen to minimize the effect that differences in GIC might have on the results, while noting that QTL positioned at the telomere itself (0 cM) would be likely to suffer from lower detection rates due to lower information contents typically observed at the telomeric extremes. The centromeric position (49 cM) was selected for study as it is known that the rate of double reduction typically falls to zero at the centromeres (Bourke *et al.* 2015).

The QTL analysis and setting of significance thresholds was performed as described above (although permutation tests were now based on 2500 permutations, one of each phenotype set, with α = 0.05 as before). We also wished to investigate the rate at which the QTL segregation type and mode of action was correctly reconstructed. Hackett *et al.* (2014) describe a QTL model-selection method by fitting the 36 phenotype means at the QTL peak and comparing the Bayesian Information Criterion (BIC) (Schwarz 1978) for SxN, DxN and SxS models.

If the QTL model is fit using ordinary linear regression, the BIC is given by the formula:

$$BIC=n*\log\left(\frac{RSS}{n}\right)+p*\text{log}(n)$$

where *p* is the number of parameters in the QTL model, *n* is the number of data points (either 36 or 100 for the noDR and DR models, respectively) and *RSS* is the sum of squared residuals from the linear model. In our approach, *p* = 2 in the case of additive or dominant QTL (Supplementary Table 1), while p≥2 in the study of multi-allelic QTL, depending on the number of QTL alleles (Supplementary File S3).

Whereas Hackett *et al.* (2014) restricted their model search to three QTL segregation types, we expanded the search to include all possible bi-allelic QTL models (either additive or so-called “simplex” dominant, *i.e.*, a single dominant allele causes full trait expression (Rosyara *et al.* 2016)), comprising in total 224 different models (listed in Supplementary Table S1).

### Hexaploid study

Given the higher complexity of autohexaploid inheritance we restricted our study to a smaller parameter set (Table 1), with 10 populations of population size 200 for each level of multivalent pairing (0, 0.5 and 1) simulated in PedigreeSim, and 10 traits simulated per population as described above. QTL were simulated to be bi-allelic, positioned randomly across the chromosome and with random phase configuration (in the case of dominant QTL, only segregating QTL types were considered). We tested both a low-heritability (h^2^ = 0.1) and high-heritability (h^2^ = 0.7) scenario. QTL detection power and precision were investigated using the methods already described.

### Single-marker analysis

To provide a comparison between IBD-based and single-marker based approaches, we also ran a single-marker ANOVA using the raw marker dosage scores as the explanatory variable, *i.e.*

Y=μ+aD+ε

where *Y* is the vector of phenotypes, *D* the vector of marker dosages (missing values removed), μ the intercept and ε the residuals.

### Multi-allelic QTL

A common simplification is to assume QTL are bi-allelic. However, it may be possible that multiple functional alleles exist at a QTL locus. We therefore also performed a separate simulation study to investigate the performance of our QTL mapping approach with multi-allelic QTL. For conciseness, this was performed in tetraploids only (Supplementary File S3).

### Application to real data

The *Altus* x *Colomba* (AxC) tetraploid potato mapping population (Bourke *et al.* 2016) was used to explore both QTL models and test the methods described earlier for simulated data. The genetic positions of 6912 SNP markers from the SolSTW 20K SNP array (Vos *et al.* 2015) were taken from a previously-published high-density linkage map developed using this population (Bourke *et al.* 2016). A subset of these markers was selected as input data for TetraOrigin (Zheng *et al.* 2016). Markers were selected so that each consecutive 0.5 cM window had (where possible) one marker of every segregation type (in a tetraploid there are nine (Bourke *et al.* 2016)), selecting markers randomly among those with fewest missing values. TetraOrigin IBD probabilities computed under the assumption of no double reduction (noDR) or allowing for the possibility of double reduction (DR) were saved for later QTL analysis (after confirming that homolog numbering between the noDR and DR datasets was consistent).

The two traits investigated were plant maturity and tuber flesh color. Both were scored on an ordinal scale, with maturity scored from 1 (very late) to 9 (very early) in increments of 1, and flesh color scored from 4 to 8 through varying shades (4 = white, 5 = cream, 6 = light yellow, 7 = yellow, 8 = dark yellow). Maturity was scored visually in the field during the growing seasons 2012, 2013 and 2014, with flesh color scored post-harvest for each of these years (three replicates). QTL analysis was performed as described in the previous sections, using splined IBD probabilities as weights in both a noDR and DR model for comparison purposes. Individual analyses per year were performed, as well as a general analysis using best linear unbiased estimates (BLUEs) generated using the lme function from the nlme R package (Pinheiro *et al.* 2017) with Year as random effect and genotype as fixed effect. QTL were re-mapped by saturating the LOD-5 support intervals around the QTL peaks (no marker binning performed) and re-estimating IBD probabilities in TetraOrigin. QTL analysis was subsequently performed at the marker positions themselves (rather than at splined positions) to better estimate the peak positions. The location of the *CYCLING DOF FACTOR 1* (*StCDF1*) locus on chromosome 5 (Kloosterman *et al.* 2013) with gene annotation PGSC0003DMG400018408, and the *BETA-CAROTENE* *HYDROXYLASE 2* (*StChy2*) locus on chromosome 3 (Wolters *et al.* 2010) with gene annotation PGSC0003DMT400026363 from the potato genome sequence version 4.03 (SpudDB Genome Browser *S. tuberosum* group Phureja DM1-3 (Hirsch *et al.* 2014; Potato Genome Sequencing Consortium 2011)) were used to compare the physical and genetic positions of the QTL peaks. The most likely QTL model at the peak positions was explored by selecting the QTL model that minimized the BIC among the bi-allelic and (simplex) dominant models listed in Supplementary Table S1.

We also determined the average allele effect around the QTL positions as $\bar{h}-\bar{y}$, where $\bar{h}$ is the weighted average phenotypic score, *i.e.*, $\bar{h}=(\sum_{i=1}^{N}\pi_{i}y_{i})/(\overset{N}{\underset{i=1}{\sum }}\pi_{i})$ for IBD probabilities $\pi_{i}$ (using the noDR model) and phenotypic BLUE scores $y_{i}$ for individual *i*, and $\bar{y}$ is the overall population mean phenotypic scores (*i.e.*, overall mean of the BLUEs).

### Data availability

Supplemental material available at Figshare: https://figshare.com/s/a97475f5802e3354f44e.

## Results

### Effect of GIC on QTL analyses

**Figure 3:**
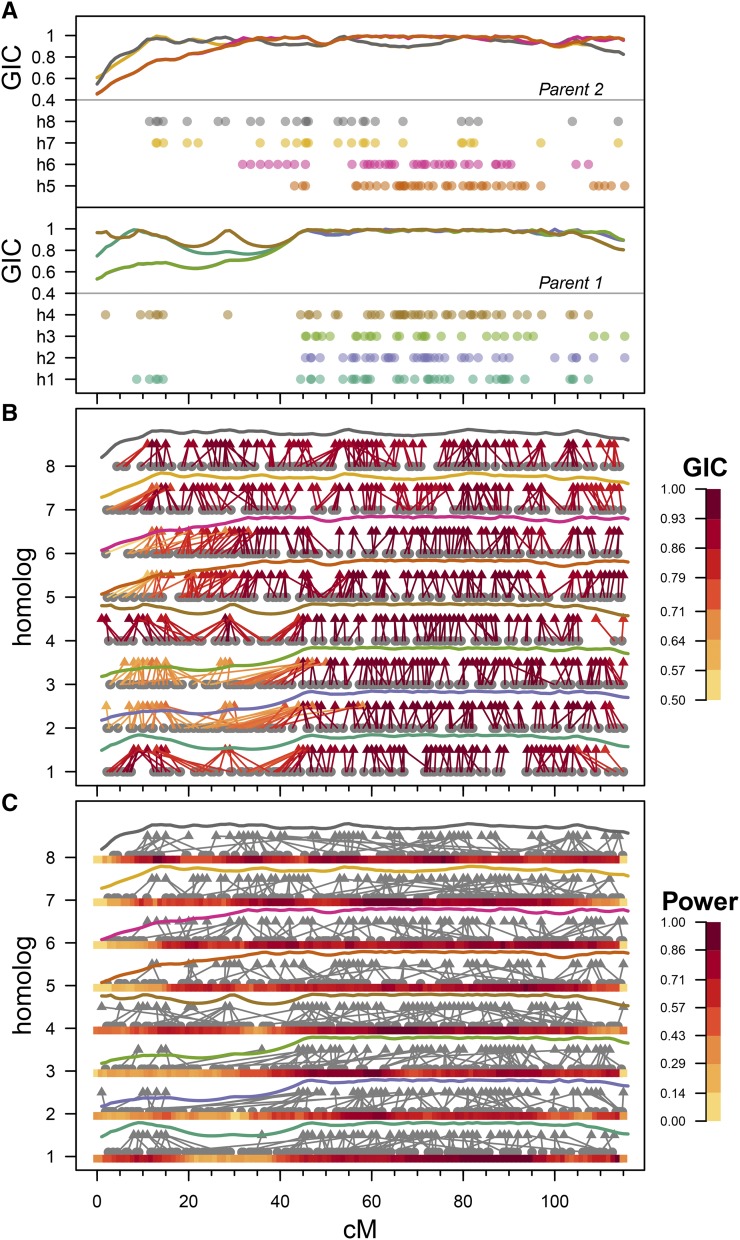
The effect of variable Genotypic Information Coefficient (GIC) explored. **A.** Influence of marker distribution on GIC values (calculated using noDR IBD probabilities) on potato chromosome 1 (Hackett *et al.* 2013), with parent 1 values in the lower panel and parent 2 values in the upper panel. Average GIC values over the 10 simulated populations are shown as lines above the marker distribution (and carry the same color). On the y-axis, h1 – h4 = parent 1 homologs 1 to 4, and h5 – h8 = parent 2 homologs 5 to 8. Occasionally GIC lines overlap, obscuring each other, although their profile logically follows the marker allele distribution shown. **B.** Effect of variable GIC on the precision of QTL detection. True QTL positions per homolog are represented by gray dots (each from a separate analysis – we did not simulate scenarios of more than one QTL per chromosome), with arrows indicating the position of the discovered QTL peak. Arrows are colored by the GIC content at the QTL position itself, with average GIC lines from Fig. 3.a shown above the arrows. The example shown corresponds to SxN additive QTL with a population size of 400, heritability of 0.2 and multivalent rate *q* = 0 analyzed using the noDR model. In this figure, all simulated QTL were detected (full power). **C.** Effect of variable GIC on the power of QTL detection, visualized on a per-homolog basis. Here, the power in a 10 cM sliding window is shown by a heat-map track below each homolog. QTL positions are shown as gray dots, with arrows indicating the position of the discovered QTL peaks. In contrast to (b), there was not full detection power (population size 200 and heritability = 0.1) – hence variation in QTL detection power along each homolog is apparent, and corresponds quite well with variations in the estimated GIC per homolog, shown above the arrows.

As expected, there was a clear relationship between the GIC per homolog and the marker coverage of that particular homolog (an example is given in Figure 3.a). Differences between coupling and repulsion marker information can be discerned, for example where a single 1x0 (SxN) marker tagging homolog 4 in parent 1 at 28.5 cM gave a large boost to the otherwise low GIC values in that region on homolog 4, but also slightly increased the GIC values on homologs 1, 2 and 3 (there is no information about the meiosis of parent 2 from such a marker). The GIC accounted for much of the variability in QTL detection power, although not as much as the population size or trait heritability (Supplementary Files S5 & S6).

Apart from the influence of GIC on detection power, we were also interested in understanding the influence of GIC on the accuracy of QTL analysis. For this we examined more closely the position of QTL peaks in relation to their true position, for SxN QTL only (since these originate from a single homolog and are simpler to track). We noted a dramatic influence of GIC on the QTL peak position in regions of variable GIC, even in situations with 100% detection power (Figure 3.b). Local maxima in GIC such as that observed in parent 1 homolog 4 at 28.5 cM serve as local “attractors” for QTL peaks, an effect seen across all homologs. Generally-speaking, there is a tapering of GIC profiles at the telomeres, a consequence of poorer marker information (coming from one side only). Where GIC is high, the true position and detected QTL peak closely corresponded (Figure 3.b, 40 – 100 cM region). A visualization of the homolog-specific variation in QTL detection power is given in Figure 3.c.

For more complex QTL types such as DxN or SxS QTL, we were curious to know whether the presence of a single QTL allele on a homolog with high GIC would be enough to detect that QTL, or whether high GIC was needed on both homologs. As described in the Methods section, we categorized QTL as either LL, LH, HL or HH depending on the per-homolog GIC underlying the QTL alleles with positive effect. As can be seen in Table 2, the detection power of LL-type QTL tended to be lower than that of LH- or HL-type QTL, which themselves tended to be detected less often than HH-type QTL. In fact, the intermediate class (having only one positive QTL allele residing on a high-GIC homolog) was detected at approximately the midpoint of the LL-type and HH-type detection rates (Supplementary Figure S2).

**Table 2:** Power of detection of complex DxN or SxS QTL, categorized by the GIC on the homologs carrying the positive QTL alleles

				*High GIC threshold = 0.9*			*High GIC threshold = 0.95*		
Pop	h^2^	QTLseg	action	LL	LH/HL	HH	LL	LH/HL	HH
200	0.1	SxS	additive	0.38 (643)	0.58 (1179)	0.75 (114)	0.48 (1204)	0.59 (716)	0.81 (16)
200	0.2	SxS	additive	0.83 (632)	0.97 (1285)	0.98 (133)	0.90 (1260)	0.98 (774)	1.00 (16)
200	0.1	SxS	dominant	0.12 (641)	0.31 (1210)	0.49 (129)	0.21 (1247)	0.33 (710)	0.70 (23)
200	0.2	SxS	dominant	0.52 (574)	0.78 (1228)	0.93 (150)	0.63 (1169)	0.83 (761)	0.82 (22)
200	0.1	DxN	additive	0.43 (947)	0.48 (710)	0.74 (415)	0.46 (1396)	0.54 (531)	0.81 (145)
200	0.2	DxN	additive	0.89 (940)	0.95 (626)	1.00 (436)	0.92 (1361)	0.95 (507)	1.00 (134)
200	0.1	DxN	dominant	0.16 (944)	0.20 (646)	0.35 (372)	0.18 (1378)	0.25 (471)	0.38 (113)
200	0.2	DxN	dominant	0.53 (985)	0.60 (678)	0.87 (373)	0.56 (1418)	0.70 (483)	0.90 (135)
400	0.1	SxS	additive	0.86 (654)	0.96 (1255)	0.98 (127)	0.91 (1261)	0.96 (756)	1.00 (19)
400	0.2	SxS	additive	1.00 (670)	1.00 (1230)	1.00 (134)	1.00 (1246)	1.00 (780)	1.00 (8)
400	0.1	SxS	dominant	0.55 (681)	0.79 (1194)	0.91 (125)	0.64 (1275)	0.84 (708)	0.71 (17)
400	0.2	SxS	dominant	0.96 (684)	1.00 (1168)	1.00 (132)	0.98 (1312)	1.00 (652)	1.00 (20)
400	0.1	DxN	additive	0.86 (946)	0.93 (683)	0.99 (415)	0.89 (1392)	0.95 (492)	0.99 (160)
400	0.2	DxN	additive	1.00 (956)	1.00 (643)	1.00 (421)	1.00 (1369)	1.00 (502)	1.00 (149)
400	0.1	DxN	dominant	0.57 (935)	0.57 (604)	0.84 (465)	0.59 (1380)	0.70 (488)	0.85 (136)
400	0.2	DxN	dominant	0.95 (900)	0.96 (611)	1.00 (393)	0.96 (1316)	0.97 (440)	1.00 (148)

QTL detection power was determined for two different definitions of “high GIC” – either exceeding 0.9, or exceeding 0.95. Results for different levels of multivalent pairing and QTL model used (DR and noDR) were combined. Numbers in brackets refer to the numbers of separate QTL on which the estimates of power are based.

Pop = Population size; h^2^ = heritability; QTLseg = QTL segregation type, where DxN denotes a duplex QTL, so either 2x0 or 0x2, and SxS implies a 1x1 marker; action = mode of QTL action. LL, LH and HH refer to QTL with alleles on homologs of both Low GIC, of Low and High GIC and of both High GIC, respectively. Results for LH and HL were considered equivalent and were combined.

Finally, we found that the GIC was influenced by the haplotypic diversity within the mapping population, with more diverse populations leading to on-average higher GIC values than in populations derived from more genetically-homogeneous material (Supplementary File S2). However, lower haplotype diversity also contributed to greater levels of homozygosity and fewer segregating markers, meaning fewer markers were available for IBD calculation in these populations.

### Power to detect QTL

As noted in the previous section, population size and trait heritability were found to have the most impact on QTL detection power, followed by GIC. On potato chromosome 12 we deliberately chose two QTL locations to minimize the impact of GIC and allow a comparison of centromeric *vs.* telomeric effects (with average cross-homolog GICs of 0.95 and 0.98 for 14 and 49 cM respectively).

**Figure 4:**
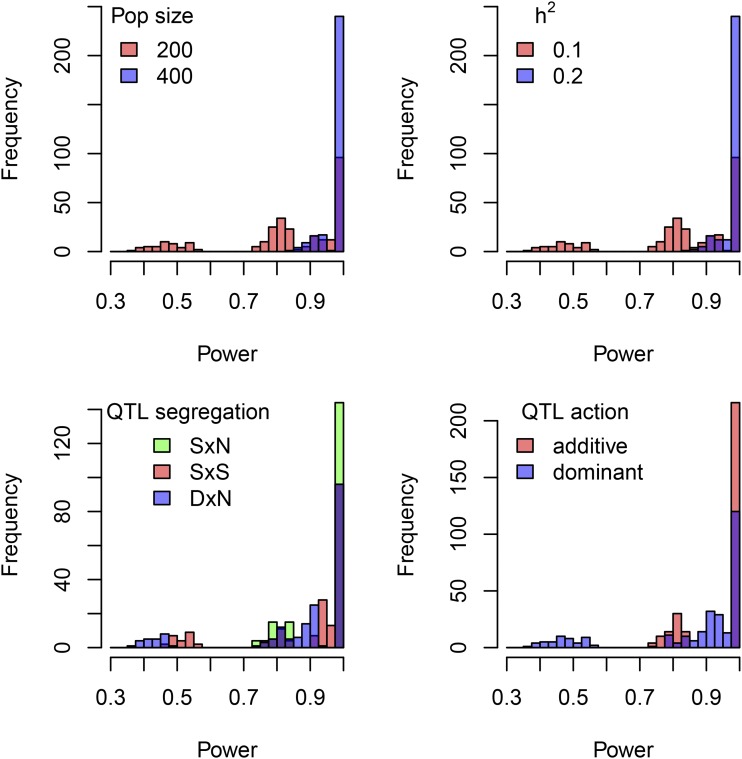
Distribution of QTL detection power, grouped by main explanatory effects. Here we show the distribution of QTL detection powers among the 576 estimated QTL detection powers we determined (*c.f*. Table 1, chm. 12 power study for the set of experimental parameters). Most scenarios had a very high detection power (> 0.95), although powers as low as 0.35 were also observed. Additive SxN QTL with a mapping population of 400 and heritability of 0.2 are likely to always be detected, whereas a dominant DxN QTL with a mapping population of 200 and heritability of 0.1 is unlikely to be detected more than 50% of the time.

**Figure 5:**
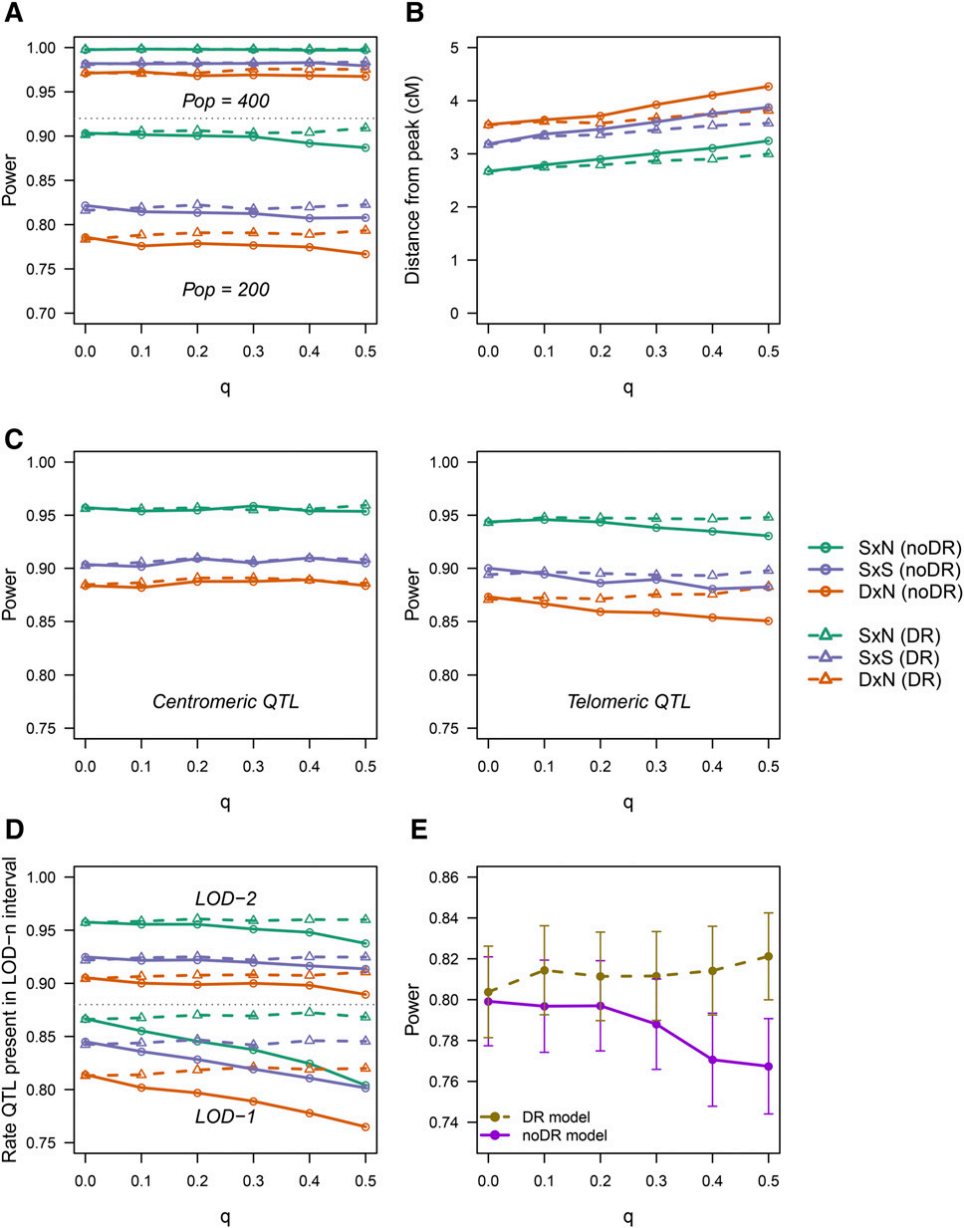
Effect of various experimental parameters on QTL power and precision. **A.** QTL detection power for population size 200 and 400 compared over a range of different rates of multivalent formation (*q*). Solid lines indicate the results using the random bivalent noDR model, with dashed lines indicating the results using the DR model (including double reduction). Results over both trait heritabilities are combined here - the comparison between lower and higher trait heritabilities showed the same trend. **B.** Effect of double reduction on QTL precision, depicted by the average distance between QTL peak and true QTL position across all experimental scenarios. **C.** Comparison between detection power for a centromeric QTL (left) and a telomeric QTL (right). **D.** Rate at which QTL fall within LOD-2 and LOD-1 intervals. **E.** Specific example of the difference in power between the DR and noDR models for the case of an additive SxN QTL with population size 200 and trait heritability of 0.1, up to 5% higher power on average when the rate of quadrivalent pairing was high. Standard errors of the means ($\frac{s}{\sqrt{n}}$) are depicted by error bars.

The four most important factors in determining QTL detection power (excluding GIC) were population size, trait heritability, QTL segregation type and QTL mode of action (Figure 4). When we ran a GLM using all the available explanatory variables we found that neither the rate of multivalent formation (*q*) nor the form of the model used (DR or noDR) had any real impact on the QTL detection power overall (Supplementary File S6). However, there were some instances where the DR model could improve detection power. For example, when the population size or trait heritability is low and rate of multivalent pairing is high, the DR model does offer an advantage (Figure 5.a), helping to maintain the same level of power as that achieved when there is strictly bivalent pairing (*q* = 0). Regarding the precision of the QTL analysis, the average distance from the QTL peak to the true QTL position is also adversely affected by double reduction (Figure 5.b). However in this instance, no matter what model is used, the QTL analysis will become slightly less precise at higher values of *q* (although there is some mitigation of the loss of accuracy when the DR model is used). Here we used distance as an absolute measure – the direction of this distance appeared to be biased toward the side of the QTL with greater genetic length (Supplementary Figure S3).

As expected, there is essentially no difference between the two models for centromeric QTL, but differences do appear for telomeric QTL (Figure 5.c). If we consider the rate at which QTL were present in the LOD-1 and LOD-2 intervals instead, we see a very sharp decline in the performance of the LOD-1 interval at higher levels of multivalent formation if the noDR model is used (Figure 5.d). However, it is questionable whether the LOD-1 interval should be used at all – even in the case of purely bivalent pairing, on average 16% of these support intervals contain no QTL – a value which increases to almost 32% at the lower rates of population size and heritability. The widths of the support intervals around QTL peaks were also found to increase as the levels of multivalent formation increased, an undesirable effect that cannot be mitigated by using the DR model (Supplementary Figure S4).

For dominant QTL, we found the detection power of simplex dominant QTL was somewhat affected by the presence of quadrivalents, but the detection of duplex dominant QTL (where two copies of the allele are required for complete trait expression) was severely compromized when quadrivalents were present (Supplementary File S3). If we remove the major sources of variation from the data by considering only an additive SxN QTL with population size 200 and heritability of 0.1, we find that the DR model has the potential to increase detection power by up to 5% when the rate of multivalent formation is high (Figure 5.e).

### Accuracy in predicting the QTL configuration and mode of action

Apart from the ability to detect QTL, we were also interested in investigating methods to correctly predict the QTL configuration (*i.e.*, predicting from which parental homologs the QTL alleles originate (also called the QTL segregation type or QTL phase)), and what the most likely mode of action is (additive or dominant). We followed the procedure described in Hackett *et al.* (2014) for this, using the Bayesian information criterion (BIC) to compare different bi-allelic QTL models. All possible bi-allelic QTL models were tested, as listed in Supplementary Table S1. In most cases, the minimum BIC corresponded to the correct QTL configuration and mode of action, with near-perfect accuracy for all QTL types in higher-power experimental designs (N = 400 and h^2^ = 0.2).

### Hexaploid analysis

The TetraOrigin algorithm (Zheng *et al.* 2016) was also implemented for autohexaploids, but due to excessive memory demands we were unable to run a fully general analysis. We therefore only considered multivalent pairing in one or other parent, but not both. In hexaploids, multivalent formation is a poorly-understood phenomenon. We took the most general approach of considering hexavalent formation, which also accounts for genotypes generated by a quadrivalent + bivalent pairing. We found that the presence of multivalents caused a slight reduction in detection power and precision (here, looking at the width of the LOD-2 support interval) which could be mitigated to some extent by using the DR model (Supplementary Figure S6.a). At the higher trait heritability (h^2^ = 0.7) we found that QTL presence in LOD-2 intervals actually dropped, although this was a result of much sharper QTL peaks and hence much narrower intervals (∼2 cM *vs.* ∼45 cM). Prediction accuracies of QTL phase and mode of action barely exceeded 10% at low trait heritability (h^2^ = 0.1). while at higher heritability, QTL phase prediction was near-perfect.

### Single-marker analyses

We also analyzed the hexaploid and (a subset of) the tetraploid data using a single-marker QTL model. The rates of QTL detection within LOD-2 intervals (defined as the maximum interval for which the LOD score differed by at most 2 from the peak LOD) ranged between 70–85% for both tetraploids and hexaploids, although these intervals were extremely wide (60 – 80 cM), covering most of the chromosome (Supplementary Figures S7 and S8 for tetraploid and hexaploid, respectively). In contrast, using the IBD model resulted in much narrower LOD-2 support intervals (Supplementary Figures S4 and S6).

### Multi-allelic QTL

We performed a smaller complementary study using simulated multi-allelic rather than bi-allelic QTL (described in Supplementary File S3). On the whole, the results are consistent with what we discovered using simple bi-allelic QTL only. For example, the presence of quadrivalents (and hence double reduction) lead to slightly lower detection powers, although if the DR model is used this effect can be offset somewhat. The number of QTL alleles present did not appear to have much impact on QTL detection power. However, we did observe an increase in prediction accuracies if the DR model was used, but only in the extreme case of complete quadrivalent pairing (Supplementary File S3).

### Application to real data

**Figure 6:**
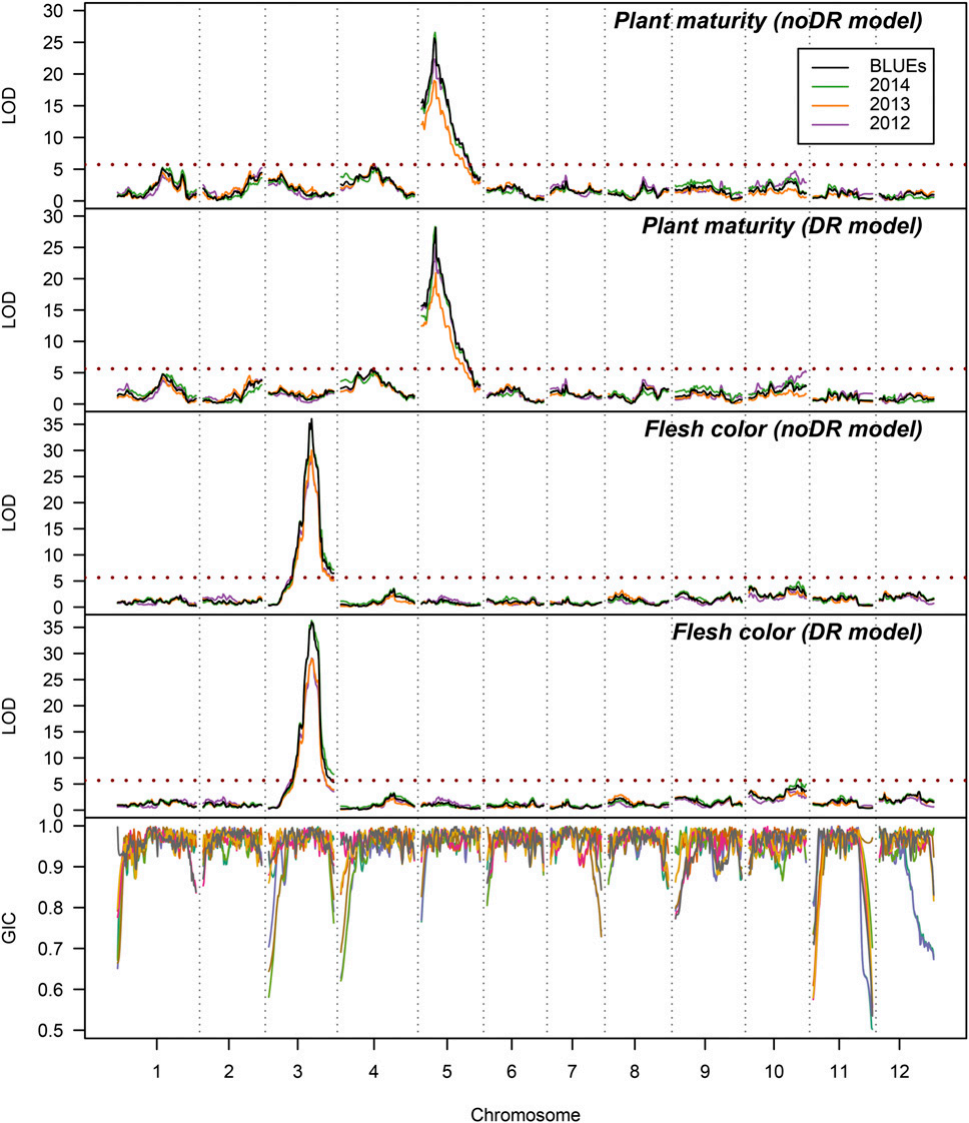
Results of the QTL scans for traits plant maturity and tuber flesh color in the tetraploid AxC potato population (N = 222 for most analyses, *c.f*. Table 3). Results using the noDR model (random bivalent) are shown above those of the DR model (allowing double reduction) for both traits. LOD significance thresholds are shown as dashed red lines. The lowest panel shows the GIC per homolog for the eight parental homologs (using the noDR IBD probabilities), using the same color scheme as Figure 3.A.

To help illustrate our findings we looked at two well-studied traits for which phenotypic data were available from the AxC tetraploid F_1_ potato population. AxC is the result of a wide cross between the late, white/cream fleshed starch cultivar *Altus* and the early, yellow fleshed ware cultivar *Colomba*. On average the rate of quadrivalent pairing was 0.24 (and was similar between parents: parent 1 = 0.23 ± 0.07 and parent 2 = 0.25 ± 0.05) (Supplementary Figure S9), consistent with a previous estimate of 0.2 – 0.3 from this population using only SxN marker information (Bourke *et al.* 2015). The phenotypic traits themselves (plant maturity and flesh color) are already genetically well-characterized, offering the opportunity to compare QTL peak positions with the physical location of the underlying candidate genes as well as an exploration of the most likely QTL models. For both traits a single major QTL was found with both the noDR and DR models (Figure 6). As can be seen from the bottom panel of Figure 6, the GIC for some homologs was quite variable (*e.g.*, chromosomes 3, 4, 11 or 12) but was overall relatively high across both parental maps.

**Figure 7:**
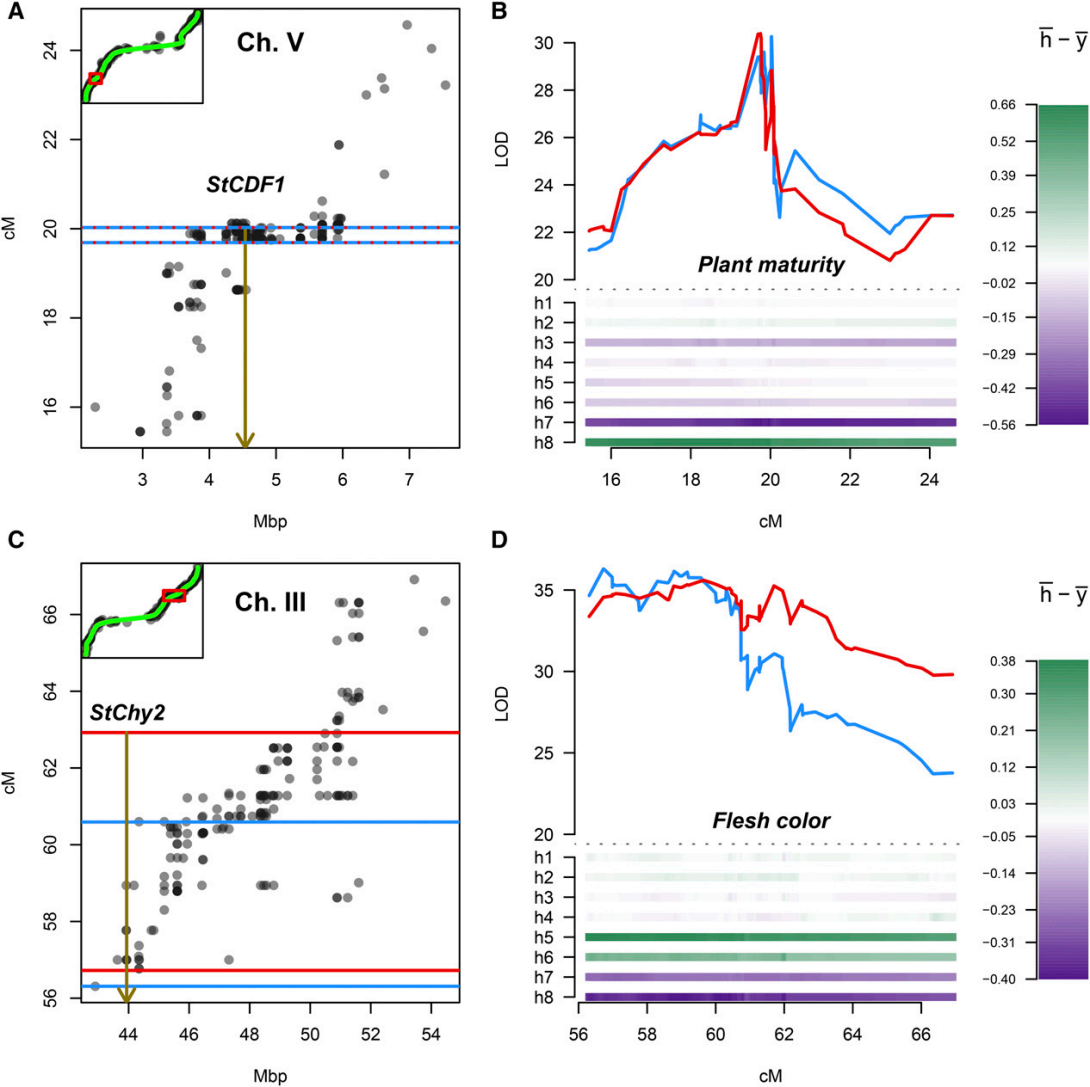
Re-mapping of the plant maturity QTL on potato chromosome 5 and the flesh color QTL on chromosome 3. **A.** Genetic *vs.* physical position of a subset of markers within the re-mapped QTL region of chromosome five (Ch. V). LOD-2 support intervals for the noDR and DR models overlapped, shown here as overlapping horizontal red / blue lines. The *StCDF1* locus is highlighted by a vertical arrow at ∼4.54 Mbp. *Inset*: Genetic v’s physical position of markers used in initial TetraOrigin analysis on chromosome 5, with re-mapped region highlighted in red. **B.** LOD profiles of the re-mapping of the chromosome 5 QTL for plant maturity. noDR model results are shown in blue, with DR model results in red. Underneath the LOD profile, the average allele effects per homolog are shown ($\bar{h}-\;\bar{y}$, where $\bar{h}$ is the weighted mean of the allele effect originating from parental homolog *h* (weighted by IBD haplotype probabilities) and $\bar{y}$ is the overall phenotypic mean). y-axis labels “h1” – “h8” represent parental homolog numbering. The range of allele effects are shown by the bar on the right. **C.** Similar to (A), showing instead the re-mapped region of the chromosome 3 (Ch. III) peak for flesh color. The *StChy2* locus is highlighted by a vertical arrow at ∼43.94 Mbp. *Inset*: Genetic v’s physical position of markers used in initial TetraOrigin analysis of chromosome 3. **D.** LOD profiles of the re-mapping of the chromosome 3 QTL for flesh color. noDR model results are shown in blue, with DR model results in red, as before.

There was a single QTL peak for plant maturity on chromosome 5 around 18 – 20 cM (Table 3). Using the DR model, slightly more of the variance was explained than with the noDR model (44% *vs.* 41%), and the width of the LOD-2 intervals was narrower (1 cM *vs.* 3 cM). When we saturated the QTL region with marker information and re-ran the IBD calculations and QTL analysis, we were able to increase the proportion of explained variance at the peak QTL position to 47%, with the peak occurring at approximately 20 cM using both models (Table 4). The most likely QTL model in a search among bi-allelic additive and simplex dominant QTL models (*c.f*. Supplementary Table S1) was an additive oooo x oooQ model, (where ‘o’ = no effect and ‘Q’ = having an effect, in this case positive *i.e.*, increasing earliness) (Table 4). When visualizing the allele effects (Figure 7.b) the “early” allele from *Colomba* appears to have been balanced by a “late” allele (darker purple), somewhat unexpectedly for an early variety. The mean maturity of offspring that inherited the early allele from *Colomba* was 6.9 ± 0.7, and without was 5.7 ± 0.9.

**Table 3:** Major QTL peaks for potato maturity (chromosome 5) and flesh color (chromosome 3) detected in AxC, phenotyped over 3 seasons (2012 – 2014)

Trait	Chm.	Year	Model	Peak cM	N	LOD	LOD-2	|LOD-2|	Var.
Maturity	5	2012	noDR	18	222	22.3	17-20	3	0.37
		2012	DR	20	222	25.1	20	0	0.41
		2013	noDR	18	221	19.0	15-20	5	0.33
		2013	DR	20	221	20.9	19-20	1	0.35
		2014	noDR	19	219	26.6	17-20	3	0.43
		2014	DR	19	219	28.3	17-20	3	0.45
		BLUEs	noDR	18	222	25.6	17-20	3	0.41
		BLUEs	DR	20	222	28.1	19-20	1	0.44
Flesh color	3	2012	noDR	60	222	29.3	57-61	4	0.46
		2012	DR	60	222	29.1	57-63	6	0.45
		2013	noDR	60	221	30.0	57-60	3	0.47
		2013	DR	60	221	29.0	57-63	6	0.45
		2014	noDR	60	219	35.4	57-61	4	0.52
		2014	DR	60	219	36.3	57-63	6	0.53
		BLUEs	noDR	60	222	36.1	57-60	3	0.53
		BLUEs	DR	60	222	36.0	57-63	6	0.53

Chm. = chromosome number; Year = year of phenotypic measurement, including best linear unbiased estimates (BLUEs) over the 3 years; Model = QTL model used, either random bivalents (noDR) or also allowing for double reduction (DR); Peak cM = position of QTL peak in centiMorgans; N = number of individuals with matching phenotypic and genotypic data; LOD = LOD score at the peak; LOD-2 = range of QTL support interval positions (loci within 2 LOD of maximum LOD); |LOD-2| = width of the LOD-2 support interval in centiMorgans; Var. = proportion of variance explained by QTL peak.

**Table 4:** Exploration of major QTL peaks

Trait	Chm.	Model	Peak cM	LOD	Var.	Phase	Act	Dir.
Maturity	5	noDR	20.03	30.3	0.47	oooo x oooQ	A	+
	5	DR	19.75	30.4	0.47	oooo x oooQ	A	+
Flesh color	3	noDR	56.72	36.3	0.53	oooo x QQoo	D	+
	3	DR	59.66	35.6	0.52	oooo x QQoo	D	+

Chm. = chromosome number; Peak cM = position of QTL peak in centiMorgans; LOD = LOD score at the peak; Var. = variance explained by QTL peak; Phase = Phasing of the QTL model that minimized the BIC at the QTL peak. “*Q*” signifies a predicted QTL allele with an estimated effect, whereas “*o*” denotes an allele with neutral effect ; Act. = Mode of gene action, in this case either (A)dditive or simplex (D)ominant models were tested; Dir. = direction of the QTL allele, either increasing (+) or decreasing (-) the trait values.

**Figure 8:**
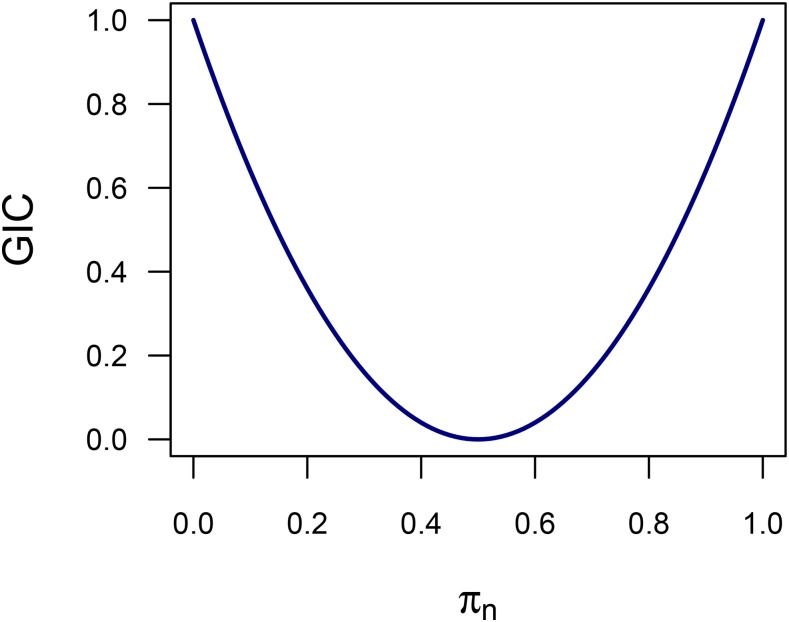
Relationship between genotypic information coefficient (GIC) and the inheritance probability of a particular parental homolog (*i.e.*, the IBD probability of inheritance of a single haplotype) in any individual *n* (π_n_)

For flesh color, the noDR and DR models resulted in the same rate of explained variance at the chromosome 3 peak (53%), with the LOD-2 interval for the noDR narrower than that for the DR model (3 cM *vs.* 6 cM), in contrast to the results for plant maturity. When we searched for the most likely QTL model, we found in both cases (noDR and DR) that a dominant model with segregation type oooo x QQoo fit the data best (Table 4). Visualizing the allele effects at the QTL confirmed that *Colomba* (the yellow-fleshed parent) most likely contributed two copies of the beta-carotene hydroxylase gene to the population (Figure 7.d).

We were interested in comparing the position of the QTL peaks with the physical location of the candidate genes *StCDF1* (for maturity) and *StChy2* (for tuber flesh color). As described in the Methods section, we saturated the LOD-5 support interval around the QTL peaks with markers and re-ran both the IBD calculations and QTL analysis. In both cases, the gene position fell within the LOD-2 intervals of the QTL peaks, although the LOD-2 interval was narrower using the noDR model, and gave a better indication of the QTL position than the DR model for flesh color; for plant maturity the support intervals from both models overlapped completely (Figure 7.c). For both traits, there appeared to be sufficient marker coverage on the important homologs within the QTL support intervals, reflected by relatively high GIC values. For plant maturity, the *StCDF1* region had far more mapped markers than elsewhere, suggesting that this locus was specifically targeted in the development of the SolSTW SNP array (Uitdewilligen *et al.* 2013; Vos *et al.* 2015). As can be seen from Figure 7.a, we were unable to separate these markers genetically due to the limited population size used for linkage map construction (N = 235), highlighting the inadequacy of this population size for fine-mapping work.

## Discussion

### The effect of variable Genotypic Information Coefficient (GIC)

Although reported as early as 1992 (Knott and Haley 1992), the influence of a variable GIC in the vicinity of QTL has essentially been ignored in many subsequent QTL studies both at the diploid and polyploid level. In this study we hope to re-emphasize its importance by demonstrating the effect of low GIC on QTL detection power, as well as the effect of variable GIC on QTL precision. According to our analysis, the GIC is one of the most important considerations in a QTL study (as well as population size and trait heritability), suggesting that dense marker coverage across all homologous chromosomes is important for successful QTL mapping.

GIC values were found to drop at the telomeres, a consequence of the one-sided information available at these regions in the multi-point IBD estimation. This has the unwanted effect of biasing the QTL detection positions inwards, making it unlikely for a telomeric QTL to be found at the correct position. This could be cause for some concern, given that telomeric regions tend to be more gene-rich than more centromeric positions. However, as telomeric regions are also known to undergo more recombination (Gaut *et al.* 2007; Li *et al.* 2015), the extent of this effect is likely to be diminished by the genetic extension of telomeric regions.

Particularly in the case of autopolyploids, knowledge of homolog-specific GIC values is helpful in predicting whether a QTL is likely to lie beneath a QTL peak, since variable GIC profiles can lead to a significant bias in the estimated QTL position. We were unable to model homolog-specific GIC in the context of double reduction, as it was not obvious how GIC should behave when an offspring can inherit more than one copy of part of a particular homolog. This could also be seen as an advantage of using the noDR model, where a homolog-specific GIC is a clearly-defined concept (Appendix 1).

The GIC cannot be further increased by increasing mapping population sizes (which is often thought to be the only way to increase power), above the limitations imposed by marker density, distribution and informativeness. If GIC values are found to be low on certain homologs, it could be worthwhile to develop more markers within that region on the affected homologs. In scenarios where this is impossible (*e.g.*, due to long stretches of homozygosity across homologs), the investigator remains blind to any potential QTL within that region, although it could be argued that such regions are unlikely to harbor segregating QTL either. For complex QTL types with more than one positive allele contributing to the trait, our results show that it is preferable to tag all QTL alleles through nearby informative markers rather than just one, or none. We also found evidence that haplotype diversity contributes to higher GIC values, although this was likely to be due to the higher number of segregating markers available in an F_1_ cross when parents are on-average less related (containing greater haplotype diversity). This implies that parents of a mapping study should be carefully chosen to maximize haplotype diversity, particularly considering that haplotype-poor population types such as F_1_ populations still predominate in polyploid mapping studies.

### The merits of a complete polysomic model

We initiated this study to determine whether it is worthwhile to include double reduction in a QTL model. Through a large simulation study we have demonstrated that there are some improvements to QTL analysis, but they are relatively minor. We saw at most a 5% increase in detection power for “low-power” situations (population size 200 and trait heritability of 0.1) when a significant proportion of multivalents are formed (*q* = 0.5). In practice however, one is unlikely to encounter rates of multivalent formation this high (Bourke *et al.* 2015). Double reduction introduces both incompatible genotypes (*e.g.*, a dosage of “2” from a SxN marker) as well as altered but otherwise credible genotypes (*e.g.*, a dosage of “2” from a SxS marker, which appears as if the marker allele was inherited from both parents, whereas in fact it came from only one). In an analysis which ignores double reduction (noDR), some noise is inevitably introduced in the IBD probabilities of certain individuals. However, this noise is essentially random across the population and the genome. On a per-individual basis the haplotypic reconstruction in the affected region can be dramatically altered. With a very limited population size (< 50 individuals) this might be problematic, but in a population of 200 or 400 individuals there is generally ample QTL signal to drown out this noise. There is no huge computational burden to running both analyses and comparing results, and this would seem to be the preferred strategy. The time-limiting and computationally-intensive step is the calculation of parental marker phase in TetraOrigin, which is arguably a redundant step given current linkage mapping methodologies which also determine parental marker phase (Bourke *et al.* 2018; Hackett *et al.* 2017).

### Investigating dominance effects in a polyploid

One of the aspects that we tried to include in our study was dominance effects, by simulating complete dominance of QTL and checking whether such QTL could be accurately detected and predicted / diagnosed. However, the topic of dominance effects, more generally termed inter-allelic interactions, becomes rather complicated in a polyploid species with multiple functional QTL alleles acting at a locus. Kempthorne described the partitioning of QTL effects into a population mean, the additive main effects of each allele and the non-additive effects composed of first-order (di-allelic), second-order (tri-allelic) and third-order (tetra-allelic) interactions (Hackett *et al.* 2014; Hackett *et al.* 2013; Kempthorne 1957). We tested whether a first-order interaction model increased our detection power for dominant QTL, but found the power was reduced slightly in comparison to the main effects model (data not shown). We therefore only included main effects in the initial QTL detection scan, which was previously recommended as a robust strategy for the initial detection of QTL effects (Hackett *et al.* 2014). Non-additive effects have also been shown to influence traits in autopolyploid populations, and such variation can be captured in a breeding program if treated appropriately (Endelman *et al.* 2018). However, we have confirmed in this study that dominant QTL can be detected at a relatively high rate using Kempthorne’s main-effects model (in fact, this appears to be the optimal detection strategy). Complete dominance (either simplex-dominant or duplex-dominant (Rosyara *et al.* 2016)) is distinguishable from additivity, but may not be the most realistic model for quantitative traits. Therefore, although we have attempted to include dominance effects in our study, we realize that this topic is probably more complex than presented here and merits further investigation.

### Diagnosing QTL segregation type and mode of action

One of the main advantages of IBD-based QTL analyses over current single-marker approaches is the ability to determine the QTL configuration and mode of action. There was little difference in the performance of the BIC model selection procedure if double reduction was included or not, but we did find the DR model performed better in the case of complete multivalent pairing (Supplementary File S3). However, this is hardly a realistic scenario biologically-speaking (Ramsey and Schemske 2002). We also found the accuracy of the procedure decreased greatly if we compared more potential QTL models (*e.g.*, testing only bi-allelic models (Figure S5) outperformed testing for multi-allelic models (Supplementary File S3), while tetraploid testing outperformed that of hexaploids (Figure S6)). This is to be expected – the more models that are compared, the greater the chance of finding an incorrect model as the most likely. However, this leads to a conundrum. There is quite a high likelihood (particularly at higher ploidy levels) of having more than two distinct functional alleles at a single locus. Restricting the model search procedure to a subset of models (as is done for example in the TetraploidSNPMap software (Hackett *et al.* 2017)) might lead to the incorrect model being chosen (simply because the correct model was not tested). On the other hand, if too many models are simultaneously compared, the overall accuracy drops.

When we tested the BIC approach using a real potato dataset we found that a search among a restricted set of bi-allelic models gave a clearer result (whether a single model or multiple models emerged as plausible) than a wider search encompassing vast numbers of multi-allelic QTL models (data not shown). However, the visualization of allelic effects (Figure 7 b&d) can at times be misleading. With simulated data we found that “positive” alleles usually appear to be balanced by one or more “negative” alleles (and vice versa), even though the remaining alleles were simulated to have *no* effect on the phenotype. QTL detection methods confined to a single genetic background such as an F_1_ population have no reference against which to objectively compare allele effects and therefore both the presence and absence of a “strong-effect” QTL allele will have an apparent contribution. With a single functional allele one would expect this balancing effect to be spread evenly over the remaining alleles of that parent (three in a tetraploid). In practice, due to random deviations from the expected segregation of allelic combinations, a different picture can emerge.

### Future outlook

In this study we have investigated QTL detection models within the context of bi-parental F_1_ mapping populations only. The relative merits of QTL mapping *vs.* genome-wide association studies are already well-documented, *e.g.*, (Korte and Farlow 2013), with bi-parental populations capturing limited allelic diversity and having low resolution due to a lack of recombination events. Multi-parental populations such as MAGIC populations (Cavanagh *et al.* 2008), or mapping using pedigree information in connected populations (Bink *et al.* 2012) offer some of the advantages of both methods, sampling a greater allelic diversity while benefitting from the extra power that comes from a more balanced population structure. Extending the methodology for IBD probability estimation to these population designs would enable more advanced QTL detection approaches to be implemented in autopolyploids. They may also provide a clearer contrast for the estimation of genetic effects, or enable the investigation of QTL effects in different genetic backgrounds. Furthermore, the impact of double reduction in wider germplasm could be investigated along similar lines to those employed here. IBD information could also be used for other applications, *e.g.*, improved estimation of complex trait heritabilities (Evans *et al.* 2018).

Genomic prediction has also been advocated as a strategy to accelerate genetic gain in autopolyploid breeding (Cellon *et al.* 2018; Endelman *et al.* 2018; Hamilton and Kerr 2018; Slater *et al.* 2016). One of the topics currently under investigation is how to optimize marker set selection for use in genomic prediction (Ma *et al.* 2016; Sousa *et al.* 2019). In autopolyploids (and to a lesser extent, in outcrossing diploids), marker coverage exists in two dimensions – along a chromosome, and across homologs of that chromosome. Here we have shown how the GIC quantifies how much information is captured by marker data across both these dimensions. It could therefore also represent a useful measure to optimize in the selection of marker sets for genomic prediction, assuming that maximizing GIC might also maximize prediction accuracy where marker number and distribution is concerned. We also did not attempt to define what constitutes a sufficiently-high GIC for QTL detection. These topics fell outside the scope of the current study, but would be interesting avenues for future research.

## Appendix 1

### Derivation of expression for Genotypic Information Coefficient, GIC

Van Ooijen (2009) described a procedure to decompose the variance associated with a QTL (*V_Q_*) into that which is explained by the markers (*V_M_*) and a residual variance for which uncertainty remains (*V_R_*). The GIC is then defined as (Van Ooijen 2009):

GIC=VMVQ=1−VRVQ

We consider the case of the GIC for one homolog of an autopolyploid, and limit our attention to the assumption of random bivalent pairing (noDR model). The derivation of the GIC per homolog when double reduction is admissible is less intuitive and has been omitted here.

At a given locus, we assume we have already calculated the IBD probability of inheritance of homolog *j*. This can be performed using TetraOrigin (Zheng *et al.* 2016) or some alternative method (*e.g.* (Bourke 2014; Hackett *et al.* 2013)). For tetraploids, 1≤j≤8 while in hexaploids, 1≤j≤12 *etc*. We can consider the IBD probability of inheritance of a particular homolog to be the inheritance probability of the alternative allele in individual *n* ($\pi_{n,a}$), with the corresponding probability of inheritance of the reference allele $\pi_{n,r}=1-\;\pi_{n,a}$.

Using the definition of variance as Var(X) = E[X^2^] – (E[X])^2^_,_ and using a mean of +1 for the reference allele and -1 for the alternative allele, the residual variance ($V_{R})$ is given by:

$$V_{R}=\frac{1}{N}\sum_{n=1}^{N}(\pi_{n,r}{\left(1\right)}^{2}+\pi_{n,a}{\left(-1\right)}^{2}-{\left(\pi_{n,r}(1)+\pi_{n,a}(-1)\right)}^{2})$$

$$\Rightarrow V_{R}=\frac{1}{N}\overset{N}{\underset{n=1}{\sum }}((\pi_{n,r}+\pi_{n,a})-{\left(\pi_{n,r\;}-\;\pi_{n,a}\right)}^{2})$$

and since $\pi_{n,r}+\pi_{n,a}=1$,

$$V_{R}=\frac{1}{N}\overset{N}{\underset{n=1}{\sum }}(1-{\left(2\pi_{n,r}-1\right)}^{2})$$

$$\Rightarrow V_{R}=\frac{1}{N}\overset{N}{\underset{n=1}{\sum }}2\pi_{n,r}(2-2\pi_{n.r})$$

$$\Rightarrow V_{R}=\frac{4}{N}\overset{N}{\underset{n=1}{\sum }}\pi_{n,r}(1-\pi_{n,r})$$

where the sum is over *N* individuals in the F_1_ population. If we assume that the QTL is not under selection so that the expected proportions of the reference and alternative allele are both 0.5, then the total variance is simply 0.5(1)^2^_+_ 0.5(-1)^2^ = 1, and therefore the genotypic information content for homolog *j* is given by

$$GIC_{j}=1-\frac{4}{N}\overset{N}{\underset{n=1}{\sum }}\pi_{n,r}(1-\pi_{n,r})\;$$

However, it should be noted that substituting for $\pi_{n,r}$ in this equation using $\pi_{n,r}=1-\pi_{n,a}$ yields an identical formula (as a function of $\pi_{n,a}$), and therefore without loss of generality we see that

$$GIC_{j}=1-\frac{4}{N}\overset{N}{\underset{n=1}{\sum }}\pi_{n}(1-\pi_{n})\;$$

This is a quadratic function of the IBD probabilities for the inheritance of homolog *j* (averaged over a population of size *N*), taking a minimum (per individual) when the IBD probability equals 0.5 (Figure 8).
